# Metabolic insights and background from naturally affected pigs during *Streptococcus suis* outbreaks

**DOI:** 10.1093/tas/txad126

**Published:** 2023-11-06

**Authors:** Lluís Fabà, Virginia Aragon, Ralph Litjens, Núria Galofré-Milà, Mariela Segura, Marcelo Gottschalk, John Doelman

**Affiliations:** Trouw Nutrition R&D, Amersfoort 3811 MH, The Netherlands; Unitat mixta d’Investigació IRTA-UAB en Sanitat Animal. Centre de Recerca en Sanitat Animal (CReSA), Campus de la Universitat Autònoma de Barcelona (UAB), Bellaterra, 08193, Catalonia, Spain; IRTA. Programa de Sanitat Animal. Centre de Recerca en Sanitat Animal (CReSA), Campus de la Universitat Autònoma de Barcelona (UAB), Bellaterra, 08193 Catalonia, Spain; OIE Collaborating Centre for the Research and Control of Emerging and Re-Emerging Swine Diseases in Europe (IRTA-CReSA), Bellaterra, Barcelona, Spain; Trouw Nutrition R&D, Amersfoort 3811 MH, The Netherlands; Unitat mixta d’Investigació IRTA-UAB en Sanitat Animal. Centre de Recerca en Sanitat Animal (CReSA), Campus de la Universitat Autònoma de Barcelona (UAB), Bellaterra, 08193, Catalonia, Spain; IRTA. Programa de Sanitat Animal. Centre de Recerca en Sanitat Animal (CReSA), Campus de la Universitat Autònoma de Barcelona (UAB), Bellaterra, 08193 Catalonia, Spain; OIE Collaborating Centre for the Research and Control of Emerging and Re-Emerging Swine Diseases in Europe (IRTA-CReSA), Bellaterra, Barcelona, Spain; Faculty of Veterinary Medicine, Swine and Poultry Infectious Disease Research Centre, University of Montreal, Saint-Hyacinthe, QC, Canada; Faculty of Veterinary Medicine, Swine and Poultry Infectious Disease Research Centre, University of Montreal, Saint-Hyacinthe, QC, Canada; Trouw Nutrition R&D, Amersfoort 3811 MH, The Netherlands

**Keywords:** immunoglobulins, meningitis, minerals, nursery pig, *Streptococcus suis*

## Abstract

*Streptococcus suis* (***S. suis***) is an endemic zoonotic pathogen still lacking adequate prevention in pigs. The present case study looked back to the occurrence and consequences of *S. suis* outbreaks in our swine research facilities in search of new metabolic and physiological insight. From a series of outbreaks, a dataset was created including 56 pigs sampled during disease detection based on clinical signs. Pigs suspected with *S. suis* infection were defined as diseased (*n* = 28) and included pigs defined as neurologically diseased (*n* = 20) when severe neurological signs (central nervous system dysfunctions, i.e., opisthotonos, ataxia, and generalized tremor) were observed. Another set of 28 pigs included respective pen mates from each case and were defined as control. Representative deaths were confirmed to be caused by *S. suis.* Tonsillar swabs were collected and analyzed by quantitative polymerase chain reaction (**qPCR**) for total bacteria, total *S. suis*, and *S. suis* serotypes (**SS**) 2 (and/or 1/2) and 9. Blood and sera were analyzed to quantify blood gases, minerals, and *S. suis* reactive immunoglobulins against current isolates. Data collected included litter sibling associations, birth and weaning body weight (**BW**), and average daily gain (**ADG**) 7 d after the disease detection. In general, the disease increased pH, sO_2_ and the incidence of alkalosis, but reduced pCO_2_, glucose, Ca, P, Mg, K, and Na in blood/serum compared to control. The SS2 (and/or SS1/2) prevalence was significantly (*P* < 0.05) increased in neurologically diseased pigs and its relative abundance tended (*P* < 0.10) to increase in tonsils. In contrast, the relative abundance of total *S. suis* was lower (*P* > 0.05) in diseased pigs than control pigs. Levels of *S. suis* reactive IgG2 were lower, but IgM were higher (*P* < 0.03) in neurologically affected pigs compared to control. Furthermore, there was an increased proportion of sibling pigs that were diseased compared to control. In conclusion, our results evidence that naturally affected pigs were associated to average performing pigs without any predisease trait to highlight but a sow/litter effect. Besides, neurologically affected pigs had increased *S. suis* (SS2 and/or 1/2) prevalence and relative abundance, a respiratory alkalosis profile, and mineral loss.

## Teaser

Naturally diseased pigs during *Streptococcus suis* outbreaks appear to be average producing pigs having no predisease condition other than a litter association. Neurologically diseased pigs were associated to a loss of minerals and a respiratory alkalosis.

## INTRODUCTION

*Streptococcus suis* (***S. suis***), is an endemic pathogen that causes severe clinical signs in young pigs. Typically, the disease outbreaks occur between 4 and 10 weeks of age with a wide range of mortality (0.5%–20%) in the absence of antibiotics characterized by cases of bacteremia that can mainly lead to central nervous system dysfunctions associated to meningitis. *S. suis* infections can also cause septicemia, arthritis, endocarditis, and sudden death (Cloutier et al., 2003; Gottschalk and Segura, 2019). *S. suis* infections are mainly associated with respiratory tract mucosa and tonsil colonization as entry (Segura et al., 2016), although gastrointestinal infection and intestinal lining translocation have been reported as possible (Swildens, 2009). While knowledge about the disease is increasing, the pathogenesis and natural infection process are not fully understood, and a repeatable model that mimics the first steps of the disease is still lacking.

Currently, 29 *S. suis* serotypes (**SS**) are identified based on capsular polysaccharide (**CPS**) antigens while most common *S. suis* serotypes causing disease in Europe, and more precisely in the Netherlands, are SS2 and SS9 (Rieckmann et al., 2020). Pigs are colonized at birth, and the transmission can occur before and after weaning when they are mixed with other litters or during outbreaks (Torremorell et al., 1998; Berthelot-Hérault et al., 2001). Outbreaks in commercial conditions are frequently accompanied with coinfections and stress factors identified as potential triggers (Segura et al., 2020), but repeatable preconditioning factors are unclear. It is unknown whether naturally infected pigs differ in the status of opsonizing antibodies compared to siblings or healthy pen-mates. Passive maternal immunity may protect progeny, but results are also controversial (Rieckmann et al., 2020). In fact, evidence indicates a clearance of *S. suis* maternal antibodies before weaning regardless of antibody level from vaccinated sows or carriers (Corsaut et al., 2021). Vaccination of suckling and/or nursery pigs (i.e., weeks 1 and 3) do not produce a protective immune response, likely, explained by inhibitory effects from maternal antibodies (Baums et al., 2010). *S. suis* is an encapsulated pathogen, and its CPS protects the bacterium against immune system clearance by phagocytic cells, thus allowing *S. suis* systemic dissemination (Roy et al., 2015). This natural resistance of *S. suis* is overcome if highly opsonic antibodies recognizing surface-exposed bacterial components, or the CPS itself, are present. The isotype profile of produced antibodies has been reported to be important when evaluating protection against *S. suis*, as this would be linked to the capacity of certain isotypes to induce opsonophagocytosis, while other isotypes are supposed to be poorly opsonic (Li et al., 2007). Nevertheless, the functionality of the different IgG subclasses in the swine species remains to be fully elucidated (Butler et al., 2009).

*S. suis* is highly present in the oral cavity of the pig. Murase et al. (2019) showed that *Streptococcus* spp. was found in the saliva microbiota at 16.9% (50.1% *S. suis*) in suckling pigs, 18.2% (51.8% *S. suis*) post-weaning, 19.4% (29.6% *S. suis*) in grower-finishing pigs and 9.9% (62.6% *S. suis*) in sows. It has been suggested that macrophages in tonsillar lymphoid tissue might act as reservoirs for replication of *S. suis* virulent strains (Segura et al., 2020). When bacterial competition in tonsils is reduced, *S. suis* could increase and escape into the bloodstream where expresses virulent factors and finds portals of entry to the brain via blood-cerebrospinal fluid (**CSF**) barrier and brain blood barrier (**BBB**). Recently, differences in tonsillar bacterial community composition, taxa, and phylogenetic diversity were reported depending on the *S. suis* health status (Niazy et al., 2022). Tonsil microbiota of both healthy and diseased pigs was dominated by *Streptococcus* amplicon sequence variants, but disease did not provide a clustering effect of different community types.

Neurological signs are the most easily recognized consequences from meningitis and streptococcal disease in swine. The inflammatory response in meninges is characterized by a reduced glucose content and increased lactate in CSF, which is specific for bacterial, but not viral, meningitis (Abassi et al., 2021). Inflammation in the blood–CSF barrier and (or) BBB is followed by a cascade of cytokines, infiltration of immune cells, and free-radical release, which prompts increased vascular permeability, inflammation, and edema (Prager et al., 2017). Recently, a delivered and controlled infection with *S. suis* under anesthesia showed a drop of pH in the CSF during infection, which correlated with cell and bacterial numbers in CSF (Buhr et al., 2021). Meningitis increases anaerobic glycolysis and lactate production in BBB adjacent cerebral tissue by CSF pleocytosis and cellular infiltrates from inflammation in the leptomeninges (Abassi et al., 2021). Lactate in CSF is reported to increase about four to eight times (Sears et al., 1974; Guerra-Romero et al., 1992; Filho et al., 2014), which drops the pH and extensively compromises the precise ionic microenvironment within the neuropil and disrupts electrical signaling and brain function (Prager et al., 2017). CSF is poorly buffered and increasing amounts of lactate are compensated best with ion exchange molecules such as CO_2_ that easily cross the BBB (Weyne and Van Leuven, 1973). Hence, the physiological response during bacterial meningitis is an increased respiratory rate and reduce pCO_2_ from the blood as described in rabbits (Sears et al., 1974). Furthermore, ion exchange compromised during meningitis may affect mineral homeostasis systemically (Bettinelli et al., 2012). Hyponatremia and hypocalcemia have been associated with worsen outcomes and recovery in children with meningitis (Conner and Minielly, 1980; Baines et al., 2000; Chao et al., 2008). There is little reported about metabolic status and minerals homeostasis during meningitis disease and even less is known in pigs. However, the study of metabolites and health status under disease conditions brings value to identify key factors and potential management, nutrition, and veterinary intervention for pig resilience (Putz et al., 2019; Dervishi et al., 2023). Therefore, it is of interest to better understand pig metabolic status around the natural occurrence of streptococcal disease.

The general objective of this study was to study a series of *S. suis* outbreaks in our swine research center (SRC, Trouw Nutrition, Sint-Anthonis, The Netherlands) and evaluate the metabolic status, immune status, and tonsillar load of *S. suis* in affected pigs under field conditions.

## MATERIAL AND METHODS

### Ethical Approval

Sampling was conducted as part of the veterinary diagnose procedure in our research facilities during disease cases in accordance with the principles outlined in the European Union Directive 2008/120/EC and the and the Dutch order ‘Besluit houders van dieren BWBR0035217’ (https://wetten.overheid.nl). Animal use in this project was reviewed by the Animal Care Committee of Trouw Nutrition.

### Housing

The research farm includes around 160 productive sows (Hypor Libra; Hendrix Genetics B.V. Boxmeer, The Netherlands) organized in batches every 5 weeks, producing around 560 pigs per batch with approximately 24 d of age at weaning. Pigs are usually mixed and randomized in their respective studies. Feed research and management studies are running constantly and simultaneously in various sections including gestating sows, lactating sows and suckling pigs, nursery pigs, and optionally, a grower-finisher phase. The general conditions for sows include housing in conventional stalls (1.43 m² per crate and length 2.38 m) from weaning to 4 d after mating. Sows are moved to a dynamic gestating group with electronic feeding systems (135 maximum number of sows at 2.25m²-10% = 2.025m²/sow; concrete partially slatted floor) and fed a commercial or experimental gestation feed (Trouw Nutrition, The Netherlands) formulated above nutritional requirements (CBV, 2020). Prior to farrowing, sows at 110 d of gestation are moved to individual farrowing pens in standard commercial type crates (surface 5.21 m² of which 0.7 m² is closed; tender slats in pig area and slatted steel below the sow) including feeding trough and drinker on slats. Ropes as nesting material and various enrichment material is provided for sows and piglets. For the piglets, there is a creep area with heating lights turned on for the first week of life. Temperature in the farrowing room is kept constant at 23–24 °C. Prior to farrowing, sows are fed 2.7–3.0 kg per day of a commercial or experimental lactation diet (Trouw Nutrition, The Netherlands), depending on parity and condition. Sows have ad libitum access to water. Once sows have farrowed, feed allowance is reduced to 2.5 kg/d and then increased by 0.5 kg/d each day from day 1, until maximum intake is reached. Lights are on from 0730 to 2200. Pigs have access to a commercial creep feed from 1 week after birth (Milkiwean Precoce, Trouw Nutrition, The Netherlands). At the nursery phase, pigs may be housed in various conditions (conventional pens with *n* = 3–5 pigs/pen or electronic feeding systems with *n* = ~15 pigs/pen; fully tender slats) in group-housing and in accordance with commercial or experimental animal density requirements for the European Union Council Directive 2008/120/EC. Pigs sampled were from independent nutritional studies or spare pigs fed commercial diets and housed in the same facilities for 36 days.

The medicine preventive program for gilts includes vaccination against porcine reproductive and respiratory syndrome virus (PRRSV) (Porcilis PRRS, Merck & Co., Inc., NJ, USA), inlfluenza virus (Respiporc Flu3, Ceva Santé Animale, France), *Erysipelothrix rhusiopathiae* and porcine parvovirus (Porcilis Ery + Parvo, Merck & Co., Inc., NJ, USA), *Mycoplasma hyopneumoniae* (Ingelvac MycoFLEX, Boehringer Ingelheim International GmbH, Germany) and *Glaesserella parasuis* (Porcilis Glässer, Merck & Co., Inc., NJ, USA). For sows, the vaccination program continues with PRRSV, swine erysipelas, and parvovirus and against *Escherichia coli* and *Clostridium perfringens* to transfer passive immunity to piglets (Porcilis Coliclos, Merck & Co., Inc., NJ, USA). Furthermore, sows are treated with anthelmintic when entering the farrowing room (Fenbendazole, Zerofen 4%, Chanelle Animal Health Ltd, Irland). Piglets are treated with injectable iron (Usoferran 200 mg/mL, Serumwerk Bemburg AG, Germany) and vaccinated against edema disease by *Escherichia coli* (Ecoporc Shiga, Ceva Santé Animale, France), PRRSV, porcine circovirus type 2, and *Mycoplasma hyopneumoniae* (Ingelvac PRRSflex, CircoFLEX, and MycoFLEX, respectively, from Boehringer Ingelheim International GmbH, Germany). All medications were used according to manufacturer instructions, to our facility SOPs, and veterinary best practices.

### Animals

Samples were collected from diseased pigs and healthy pen-mates in the nursery phase. When clinical signs compatible with streptococcal disease and with special attention to meningitis signs, were observed, a standardized description of clinical signs was taken by trained technicians or the veterinarian. The pigs were classified as neurologically diseased cases when included at least one sign indicative of central nervous system dysfunction such as loss of balance, ataxia, paralysis, opisthotonos, generalized tremor, and paddling, which are known to be associated with meningitis (Gottschalk and Segura, 2019). The other cases without the meningitis-like symptoms described above were defined as other signs and as diseased pigs. These pigs went through a progression of loss of appetite and included one or various of the following: depression, reddening of skin, and lameness associated to arthritis.

### Sampling

Venous blood samples collected from the jugular vein were collected from both the clinically affected pig and a healthy pen-mate. Blood samples were taken within the 5 min from clinical sign detection and were the first samples to be collected to minimize iatrogenic changes in blood gas levels during handling. After blood sampling, one swab (eSwab art. no. 490CE, Copan Brescia, Italy) sample from tonsils was collected without using the liquid amies media supplied. The swab head was put into a cryotube, snap frozen on dry-ice, and stored at –80 °C immediately after sampling. The BW was measured at sampling moment and repeated in 7 d for both the diseased and the control pig.

### Biochemical Analysis

Fresh blood samples were analyzed in situ (<5 min after collection) using i-STAT (cartridge CG8+; Abbott Point of Care Inc. Princeton, NJ, USA) to evaluate pH, PCO_2_, PO_2_, HCO_3_ TCO_2_ sO_2_, Na, K, iCa, glucose, hematocrit, hemoglobin, and base excess (**BE**). The status of metabolic acidosis or alkalosis was defined from BE threshold in serum as < −3 mmL/L for acidosis and >+3 mmL/L for alkalosis (Berg and Meyer, 2008). Blood was also centrifuged at 2,000*g* for 10 min to collect two serum samples (stored at −20 °C and −80 °C). Sera from −20 °C was analyzed for levels of Ca, K, Mg, and P by ICP-MS (VieCuri Medical Center, Venray, The Netherlands).

### Specific Immunoglobulin (Ig) Titers Reactive to Current S. suis Isolates

Sera from −20°C was analyzed for total Ig, IgM, IgG1, and IgG2 reactive against three strains of *S. suis* isolated during this study (see section *Treatment and diagnose*). Enzyme-Linked Immunosorbent Assay (**ELISA**) were designed for total Ig, IgM, IgG1, and IgG2 and conducted in IRTA Centre de Recerca en Sanitat Animal (CReSA, IRTA-UAB, Universitat Autònoma de Barcelona, Bellaterra, Spain) following the method described by Corsaut et al. (2021). In brief, the three *S. suis* isolates were used for coating for ELISA Polysorb plates (Nunc-Immuno; Thermo Scientific, Mississauga, ON, Canada). Bacteria were grown overnight at 37 °C on plates with 5% sheep blood agar, then used to isolate colonies in 5 mL of Todd-Hewitt broth (Becton Dickinson, Mississauga, ON, Canada). Dilutions were made as 10 μL of 1/1,000 from 8-h cultures and transferred into 30 mL of Todd-Hewitt broth and incubated for 16 h at 37 °C with agitation. Stationary-phase bacteria were washed with phosphate-buffered saline (PBS) at pH 7.3. The bacteria pellet was adjusted to 10^7^ CFU/mL. Then, plates were coated with 100 μL/well with the whole bacteria suspension, air-dried during 2 days at room temperature (**RT**), fixed with methanol, let methanol evaporate, and finally plates were stored at RT until use. After washing, 100 μL of serial two-fold based dilutions of pig sera in PBS were added to each well and RT incubated for 1 h. For the titration total Ig (IgG + IgM) or IgM, plates were incubated 1 h at RT with peroxidase-conjugated goat anti-pig total Ig (IgG + IgM) (Jackson ImmunoResearch, West Grove, PA) or IgM (AbD Serotec, Raleigh, NC) antibodies. For porcine IgG1 or IgG2 detection, mouse anti-porcine IgG1 or IgG2 (BioRad, Mississauga, ON, Canada) was added for 1 h at RT. After washing, peroxidase-conjugated goat anti-mouse IgG (Jackson ImmunoResearch) was added for 1 h at RT. Plates were elaborated with 3,3,5,5-tetramethylbenzidine (InvitroGen, Burlington, ON, Canada) substrate, and the enzyme reaction was stopped by addition of 0.5 M H2SO4. Absorbance was read at 450 nm with an ELISA plate reader. For each serum and isolate, a titer was calculated as the reciprocal of the last serum dilution, which resulted in an optical density above the cutoff of each test (optical density at 450 nm of ≤0.2 as cutoff). Furthermore, an internal reference positive control was added to each plate to control variations. Such positive control comprised a pool of serum from 10 sows randomly selected in the farm. The reaction was stopped when optical density was at 450 nm of 1.0 was obtained for the positive internal control. IgG1 detection for *S. suis* 114 isolate showed repeated problems during plate coating, which was conducted three times and 19 samples showed non-detectable IgG1 as *n* = 11 from suspected *S. suis* and *n* = 8 from control pigs.

### Detection of S. suis

The DNA from the tonsillar swabs was extracted using the MagAttract PowerMicrobiome DNA/RNA EP Kit (Qiagen GmbH, Hilden, Germany) according to the manufacturer’s instructions with some modifications. Briefly, the swab was mixed in the kit’s MBL lysis buffer (without β-mercaptoethanol) including 100 µg PureLink RNaseA (Invitrogen, Thermo Fisher Scientific Inc. Hampton USA). The liquid cell suspension was centrifuged from the swab, and swab was discarded. 0.1 mm Beads from the kit were added to the sample, and bacteria were lysed for 60 s at 7000*g* using a MagNA Lyser (Roche, Burges Hill, UK). The DNA was extracted from this cell lysate using the ClearMag beads from the kit on the epMotion 5075 automated liquid handling device (Eppendorf AG, Hamburg, Germany). DNA was eluted in 200 µL of 10 mM Tris-HCl buffer (pH 8.0) and stored at −80 °C until use. To quantify bacteria, quantitative polymerase chain reaction (qPCR) method was used on the extracted DNA samples on a cfx-384 thermocycler (Bio-Rad Laboratories Inc. Hercules, CA, USA). For qPCR quantification of total bacteria, the 16S rRNA gene was targeted using the 926F (De Gregoris et al., 2011) 1027R (Srinivasan et al., 2016) primers in iQ SYBR Green Supermix (Bio-Rad Laboratories Inc. Hercules, CA, USA). *S. suis* was quantified by gene specific TaqMan-probe based qPCRs targeting genes fbpS (able to detect the 35 original serotypes of *S. suis* to include total *S. suis*-like bacteria), cps2J (SS2) and cps9H (SS9) using iQ Supermix (Bio-Rad Laboratories Inc. Hercules, CA, USA) (Dekker, 2014; Srinivasan et al., 2016). For the standard curve, 10-fold serial dilutions were prepared, from 10^7^ copies/μL until 10^1^ copies/μL, of the specific amplicons containing the target sequence. Cell numbers were calculated using the standard curve used in every individual qPCR run and expressed as Log cells/swab. For total bacteria, the mean 16S rRNA gene copy number for total bacteria from the ribosomal RNA operons database (rrnDB) (Stoddard et al., 2015) was used in the calculations.

### Treatment and Diagnose

Diseased pigs were treated according to the medication scheme as per our veterinary advise and standard operational procedures including manufacturers recommendation. For meningitis signs, the medication included ampicillin (AMPICILLIN 20% PRO INJ, Dopharma, Raamsdonksveer, The Netherlands) and dexamethasone (Dexa-ject; Dopharma). It followed as day 1: ampicillin + dexamethasone, day 2: ampicillin, and day 3: ampicillin + dexamethasone. If recovery was not complete, then medication followed as day 4: ampicillin and day 5: ampicillin + dexamethasone. Affected pigs were put in a separate hospital pen with adequate care. For each case, both sampled pigs, the diseased and the healthy pen-mate were monitored 4–5 times daily for the following 7 d. Special attention was paid to behavior, neurological signs, lameness, and swollen joints. Antibiotic use was recorded and sudden death, endpoint euthanasia, or other incidences were also recorded.

It is important to note that not all pigs with clinical signs were sampled during this study. If a pig with clinical signs was detected during the weekend or without the human resources available for adequate sampling within the 5 min after first clinical detection, then, the antibiotic medication was administered immediately as indicated by internal SOPs. Those pigs were, therefore, not included in this study (see Table 1). On the other hand, some untreated sudden death cases and euthanized pigs were used for diagnostic purposes. Necropsies were conducted in the necropsy room from the research farm and samples shipped for analysis to Wageningen Bioveterinary Research (Lelystad, The Netherlands) or pigs were sent intact to the Royal GD animal health (Koninklijke Gezondheidsdienst voor Dieren, Deventer, The Netherlands) for necropsy and diagnosis. During the approximate 1-year length of the study, at least one postmortem evaluation was conducted each outbreak and swabs from meninges, heart valves, and joints were collected for qPCR analysis and/or culture to confirm the presence of *S. suis* in tissues.

**Table 1.** Overview of cases occurred during this study with included and excluded pigs

Outbreaks	All suspected one cases with streptococcal disease	Subsample of pigs included in the study			
		All diseased	Including neurological signs	Control	Total
**First**	Other signs = 14	6	2	6	12
Batch *n* = 492	Neurological signs = 5				
Mortality *n* = 9	Sudden^2^ + acute death^3^ = 2 + 5				
**Second**	Other signs = 19	6	4	6	12
Batch *n* = 501	Neurological signs = 7				
Mortality *n* = 8	Sudden + acute death = 3 + 0				
**Third**	Neurological signs = 1	1	0	1	2
Batch *n* = 505	Sudden + acute death = 3 + 0				
Mortality *n* = 15					
**Fourth**	Neurological signs = 1	1	1	1	2
Batch *n* = 473	Sudden + acute death = 4 + 2				
Mortality *n* = 10					
**Fifth**	Other signs = 25	14	13	14	28
Batch *n* = 532	Neurological signs = 19				
Mortality *n* = 14	Sudden + acute death = 6 + 0				
	Other signs = 58	*n =* 28	*n =* 20	*n =* 28	*n =* 56
**Total**	Neurological signs = 33				
All *n* = 2503	Sudden + acute death = 18 + 7				
Mortality *n* = 56	Total *n* = 109				

^1^Not all pigs identified with clinical signs were sampled during this study. Cases detected during the weekend or without the human resources available for adequate sampling within 5 min after first clinical detection were not sampled. Then, pigs would get the antibiotic medication immediately.

^2^Sudden death includes pigs found dead without a previous clinical sign, which are not counted within mortality for pigs having neurological or other signs.

^3^Acute death includes pigs found dead after clinical signs and antibiotic medication or directly euthanized pigs used for necropsy and diagnose.

Three *S. suis* isolates from meninges, joints, and heart valves of different animals were serotyped by the agglutination test at Wageningen Bioveterinary Research (Lelystad, The Netherlands) and/or the Veterinary Medicine of the University of Montreal, using a multiplex-PCR (Montreal, Canada) following Okura et al. (2014). Multi Locus Sequence Typing (*aroA*, *cpn60*, *dpr*, *gki*, *mutS*, *recA*, and *thrA* genes) to the SS2 isolates and antibiograms were conducted to evaluate minimal inhibitory concentrations (MIC) for main antibiotics using the broth dilution method (Wageningen Bioveterinary Research Lelystad, The Netherlands). A *Streptococcus pneumoniae* ATCC strain was included as reference, this isolate is known to be sensitive to all tested antibiotics.

### Statistical Analysis

All statistical analyses were conducted using SAS v9.4 (SAS Inst. Inc. Cary, NC). Proc GLM procedure was used to evaluate differences between diseased pigs and control pen-mates by ANOVA for most of the performance parameters and the biomarkers in blood and sera. The procedure was conducted twice as two different models including main effect of disease for either 1) all suspected pigs versus control or 2) only for neurologically diseased pigs vs. control. As the group of pigs with other clinical signs was small (*n* = 8) and was heterogenous in terms of clinical signs classified, the comparison to controls and neurologically diseased pigs was not performed. Proc GLIMMIX with binomial distribution was used to evaluate acidosis or alkalosis status and to determine prevalence differences for the different *S. suis* serotypes evaluated. LSMEANS were calculated with *P*-values adjusted using simulate correction.

In addition, total Ig, IgM, IgG1, and IgG2 reactive to three the isolated strains of *S. suis* (strain 109 *S. suis* SS19, strain 114 SS2, and strain 115 SS2) were analyzed under more complex mixed models (Proc MIXED) and individual correlations and regression (Proc REG and Proc CORR). Time in the nursery and case-to-case variance at sampling, including the diseased pig and the control pen-mate, had to be included in the statistical models to evaluate immunoglobulin levels because they are known to be very variable, especially over time post-weaning. Therefore, log2 transformed total Ig, IgG1, IgG2, and IgM reactive to *S. suis* were analyzed with isolates (109, 114, and 115) as a fixed factor, the animal as repeated measure (the three samples per pig), and time at sampling as a covariate. LSMEANS were calculated with P-values adjusted using simulate correction (supplementary material). Finally, mixed regression models (Proc MIXED) were performed to study the effect of disease with neurologically diseased pigs in total Ig, IgG1, IgG2, and IgM reactive to *S. suis* isolates by regression as:

Y = Intercept + beta*Disease + beta*Time + beta*Disease*Time + error repeated measures (individual pig as three tests per pig) + error random statement (isolate) + error for residual

Y = response parameter, Ig levels in serum (log 2 transformed)

Intercept = Intercept, the expected mean value of Y when all X = 0

Beta = standardized (regression) coefficients

Disease = factor representing the status of being neurologically diseased or healthy pen-mate control (0, 1)

Time = the time in the nursery phase at sampling (detection of cases) including 17 times (4, 6, 7, 10, 11, 12, 13, 14, 15, 17, 18, 19, 20, 21, 26, 27, and 28 d as one or more case per time).

Disease*Time = the interaction between disease factor and time was removed when not significant.

Error = the errors for the isolate (109, 114, and 115) as a random effect, the animal as repeated measure representing the individual variance, and the residuals of the model.

For the bacteria and relative abundance analysis for total *S. suis* within total bacteria counts or for different serotype groups (2, 7, 9, and unknown) within total *S. suis* qPCR data, the PROC NPAR1WAY with Wilcoxon two-sample test to differentiate control vs. general disease and control vs. neurologically diseased and neurologically diseased was used.

The animal was the experimental unit for all parameters and for all analyses, *P* < 0.05 was defined as significant and *P* < 0.10 as tendency.

## RESULTS

A total of 56 pigs were sampled which included 28 pigs as suspected of *S. suis* disease with clinical signs. Among the 28 diseased pigs, 20 had severe neurological signs (defined as neurologically diseased). The incidence of cases was mainly distributed in three outbreaks and two isolated cases (Table 1). The number of days with medication averaged 3.56 times until recovery or death. Among the selected pigs in study, 4 out of 28 suspected pigs died. Two acute cases (<24 h, pigs 96508 and 91463) and two pigs that died 2 and 4 days later, respectively (pigs 96905 and 92316, respectively). During one case, respiratory rate from a neurologically diseased pig with severe neurological signs was measured at 120 respirations/minute.

From the necropsies conducted, all outbreaks were caused by SS2 (confirmed when isolated) and/or SS1/2 by qPCR (which cannot differentiate both serotypes) from sampled tissues. Neurological signs in necropsied pigs were associated to macroscopic lesions with diffuse fibrinosuppurative meningitis. In two occasions, lesions were also confirmed by histopathology by the Royal GD animal health (Koninklijke Gezondheidsdienst voor Dieren, Deventer, The Netherlands).

Three isolates were cultured from meninges, joints, and heart valves of different animals. Two isolates (isolates 114 and 115) were serotyped and confirmed to be SS2 as confirmed by Wageningen Bioveterinary Research (Lelystad, The Netherlands) and the Faculty of Veterinary Medicine of the University of Montreal (Montreal, Canada). Besides, the SS2 isolates were sequence type 1 by by Multi Locus Sequence typing (MLST). Antibiograms on these isolates determined that 114 and 115 isolates were both susceptible to penicillin (MIC = 0.01 μg mL^−1^), cephalothin (MIC = 1.0 μg mL^−1^) and trimethoprim/sulfamethoxazole (MIC = 0.2/2.38 μg mL^−1^) but resistant to erythromycin, clindamycin, lincomycin, and pirlimycin (MIC > 32 μg mL^−1^) and tetracycline (MIC = 64 μg mL^−1^). Another isolate (isolate 109) was confirmed not to be SS1/2, SS2, SS9, nor SS7 by agglutination in Wageningen Bioveterinary Research (Lelystad, The Netherlands). Indeed, this isolate was serotyped and concluded to be SS19 by PCR the Faculty of Veterinary Medicine of the University of Montreal (Montreal, Canada). The SS19 serotype was associated to a co-infection with PRRSV in lungs. There were no polyserositis findings during necropsies nor *Glaesserella* (*Haemophilus*) *parasuis* DNA found in the lesions during necropsies. Clinical signs of edema disease other than the compatible neurological signs were not reported.

### Performance and Retrospective Data

Table 2 shows the LSMEANS and the statistics for the performance parameters. Data are reported twice as 1) all suspected diseased pigs and 2) only the neurologically diseased versus control as factors. Control pen-mates and diseased pigs had similar BW and age at clinical signs detection (*P* > 0.05) and did not differ retrospectively for weaning BW or ADG between weaning and sampling (*P* > 0.05). Pigs classified as diseased tended to have reduced BW at 7 d after first sampling (*P* = 0.059), while neurologically diseased pigs had significantly reduced BW at day 7 (*P* = 0.025). The ADG was reduced 48.6% in diseased and 62.7% in neurologically diseased pigs when mortality was included (*P* < 0.001). Excluding mortality, ADG was not significantly reduced in all diseased pigs (17.7%; *P* = 0.179), while ADG in neurologically diseased pigs was 29.8% lower (*P* = 0.017). Retrospectively, a significant sibling/sow effect was observed. There was a higher proportion of siblings and a higher count of siblings within the neurologically affected group of pigs than the control (*P* < 0.02). Of note, in total, there were 40 sows 10 of which contributed 2–5 piglets (siblings) in the study, and 30 sows contributed with single origin piglets.

**Table 2.** Animal performance and sow litter background comparison between all diseased pigs and controls and only neurologically diseased and controls

	All clinical signs				Neurological signs			
	Control	Diseased	RMSE1	*P*-value	Control	Diseased	RMSE	*P*-value
Number of pigs	*28*	*28*			*28*	*20*		
Age at weaning, days	22.0	22.1	1.974	0.840	22.0	21.9	2.004	0.818
BW at weaning, kg	6.33	6.09	1.469	0.551	6.31	6.36	1.482	0.902
Age at sampling, days	38.1	37.9	6.110	0.879	38.4	36.6	5.932	0.276
BW at sampling, kg	10.2	9.65	2.429	0.445	10.2	9.71	2.253	0.461
BW after 7 days, kg	12.6	10.9	3.222	0.059	12.6	10.6	2.876	0.025
ADG^2^ including mortality, g	332	171	203.9	0.006	332	124	186.9	0.001
ADG^3^ excluding mortality, g	332	273	143.2	0.179	332	233	122.7	0.017
ADG^4^ weaning to sampling, g	249	222	74.6	0.177	248	232	67.3	0.421
Sibling^5^ yes/no, %	35.7	60.7	9.23	0.070	34.5	85.0	8.82	0.002
Sibling^6^ count (1–5)	1.82	2.32	1.480	0.212	1.79	2.85	1.453	0.016

^1^Root mean standard error.

^2^Body weight.

^3^Average daily gain between sampling (first clinical sign detection or control) to 7 days post sampling or to mortality.

^4^Average daily gain between sampling (first clinical sign detection or control) to 7 days post sampling excluding mortality.

^5^Average daily gain between weaning and sampling (first clinical sign detection or control).

^6^Pigs with siblings within the dataset were given 1 and without siblings were given 0. The variable gives the proportion of animals with siblings within Control and Diseased pigs.

^7^Sibiling count = represent number of siblings within the dataset. Pigs were given scores 1, 2, 3, 4, and 5 when having 0, 1, 2, 3, or 4 siblings, respectively. The variable gives an idea sibling associations proportion within Control and Diseased pigs.

Additional information about sow performance showed no differences between diseased or neurologically diseased and control pigs (Supplementary Table S1).

### Blood and Sera Parameters

**Figure 1.**
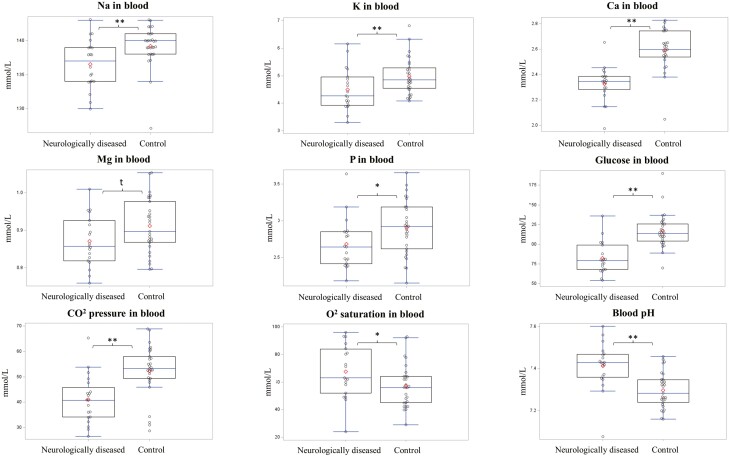
Main differences in venous blood biochemical analysis and mineral analysis in neurologically diseased pigs^1^ (*n* = 20) and control pen-mates (*n* = 28). ^1^Defined as pigs showing clinical signs of severe central nervous system dysfunctions as opisthotonos, ataxia, and generalized tremor were defined as neurologically diseased. Symbols: LSMEANS between neurologically diseased and control tended to differ *P* ≤ 0.10 (t) or differed at *P* < 0.05 (*) or at *P* < 0.01 (**).

A general pathophysiological profile was observed in blood parameters from fresh blood analyses in diseased and neurologically diseased pigs (Figure 1 and Supplementary Table S2). The profile included an increased pH, sO_2_, BE, icteric index, and an increased probability for alkalosis in the diseased pigs (*P* < 0.05). All diseased and neurologically affected pigs had significantly reduced pCO_2_, glucose, lipemia, K, Na, iCa in fresh blood and Ca, P, Mg, and K in serum compared to control pigs (*P* < 0.05). Hemolysis index was reduced in diseased pigs (*P* < 0.03), but levels of hemolysis are negligible for both classifications (3.3–7.7 index). See Supplementary Table S2 for LSMEANS and statistics.

Some outliers were removed for pig id 96508, which died very acutely (5 min) after detection of clinical signs and just before sample collection. Samples were still collected (right after death) since data had value. This case showed outlier and very low values for iCa and glucose being below average and K, Mg, and P being above average. The acute death nature of this case suggests a different pathophysiological state related to the disease. However, data from this pig are worth interpreting and were used for the discussion.

### qPCR Analysis in Tonsil Swab

Absolute total counts of bacteria and prevalence and levels of total *S. suis* and SS2 (and/or SS1/2) and SS9 in tonsil swabs are reported in Table 3. Total bacteria counts and total *S. suis* were higher (*P* < 0.03) in diseased pigs and in neurologically diseased ones relative to control pigs. Prevalence of SS2 (and/or SS1/2) in diseased pigs tended to increase (72%; *P* = 0.083), and for neurologically affected pigs significantly increased (81%; *P* = 0.039) compared to controls (44%). Tonsillar load of SS2 (and/or SS1/2) was similar in neurologically affected pigs relative to controls (*P* = 0.425). In contrast, SS9 tonsillar load was 0.69 log higher in neurologically diseased pigs than in controls (*P* = 0.008) and a similar tendency (*P* = 0.067) was observed for the analysis including all suspected diseased pigs.

**Table 3.** Tonsillar qPCR analysis between all diseased pigs and controls and only neurologically diseased pigs and controls

	All clinical signs				Neurological signs			
	Control	Diseased	RMSE1	*P*-value	Control	Diseased	RMSE	*P*-value
Total counts, log_10_ cells/swab	7.61	8.14	0.561	0.001	7.61	8.41	0.455	<0.0001
Total *S. suis*, log_10_ cells/swab	6.06	6.43	0.561	0.001	6.04	6.70	0.480	<0.0001
Prevalence of *S. suis* serotype (SS) 2 and/or SS1/2, %^2^	43.9	72.2	15.2	0.083	43.9	80.6	13.67	0.039
SS2 and/or SS1/2, log_10_ cells/swab	4.45	4.53	0.888	0.822	4.45	4.70	0.799	0.425
Prevalence SS9 yes/no, %^2^	85.6	96.3	7.058	0.198	85.6	95.0	0.070	0.327
SS9, log_10_ cells/swab	4.45	4.93	0.842	0.067	4.45	5.14	0.814	0.008

^1^Root mean standard error.

^2^Proportion of positives.

**Figure 2.**
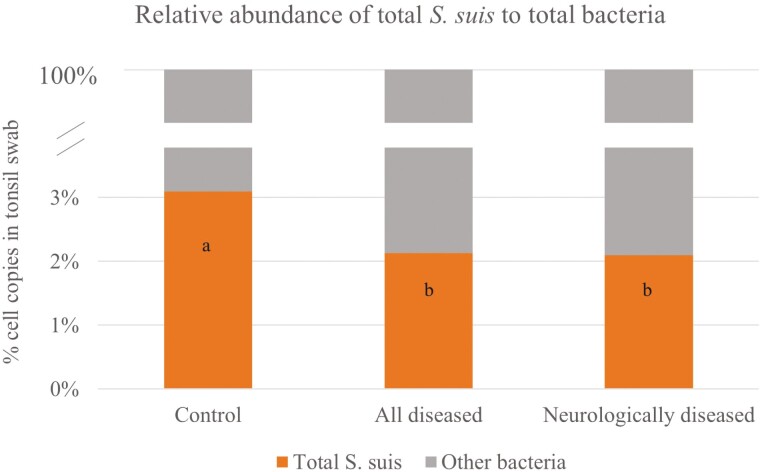
Relative abundance of total *S. suis* within total bacteria counts in tonsillar swab measured by qPCR (cells/tonsillar swab). ^a–b^ Different superscripts indicate a significant difference between categories (*P* = 0.030; Wilcoxon two-sample test).

**Figure 3.**
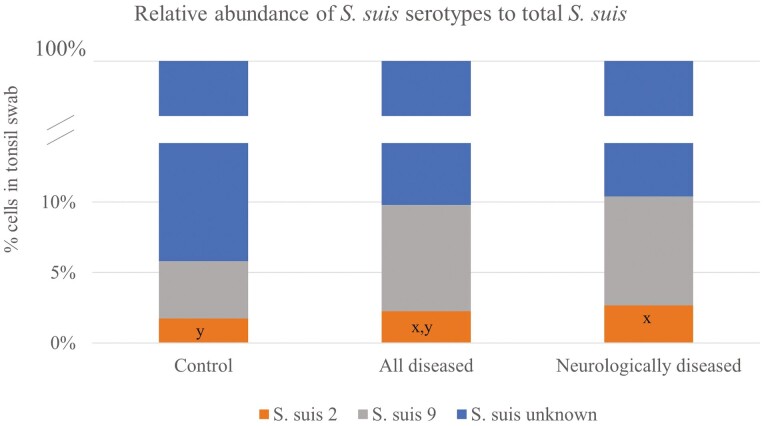
Relative abundance of *S. suis* serotypes 2 (and/or 1/2) and nine within total *S. suis* measured by qPCR (cells/tonsillar swab). ^x–y^Different superscripts showed a tendency to categories (*P* = 0.086; Wilcoxon two-sample test). *P*-values for all diseased: serotype 2 *P* = 0.192, serotype 9 *P* = 0.436, and unknown serotypes *P* = 0.476; *P*-values neurologically diseased: serotype 2 *P* = 0.083, serotype 9 *P* = 0.859, and unknown serotypes *P* = 0.867.

Looking at tonsillar data as relative abundance within total bacteria counts (Figure 2), all sick and neurologically affected pigs had lowered (*P* = 0.03) total *S. suis* relative abundance compared to controls. The relative abundance of *S. suis* serotypes within the total *S. suis* (Figure 3) showed a tendency for increased abundance in SS2 (and/or SS1/2) in neurologically diseased pigs (*P* = 0.08), but there were no effects when all diseased pigs were included or looking at SS9 (*P* > 0.10).

### Immunoglobulins Reactive to S. suis Isolates

**Figure 4.**
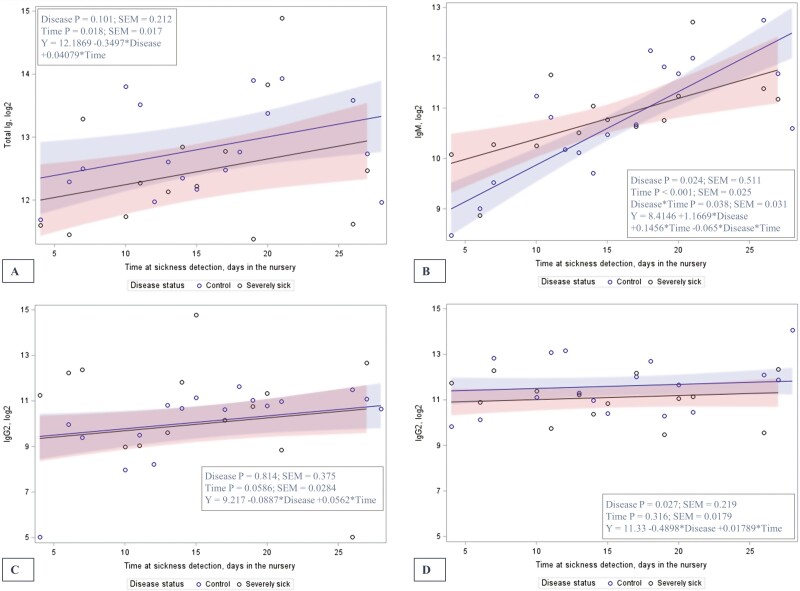
Total Ig (A), IgM (B), IgG1 (C) and IgG2 (D) levels1 in pigs either diseased^2^ as suspected of *S. suis* (*n* = 20) with neurological signs, or in control pen-mates (*n* = 28) at different times post-weaning when cases were detected in the nursery phase. ^1^Points indicate means for *n* cases on a same day post-weaning and among the values for three isolates (isolate as random effect and pig as a repeated measure). ^2^Defined as severe diseased pigs including central nervous system dysfunctions (opisthotonos, ataxia, and generalized tremor). The shadded areas indicates 95% confidence interval.

Regression mixed models were used to study *S. suis* reactive total Ig, IgM, IgG1, and IgG2 and their association with being neurologically diseased and time (Figures 4A–D). Total Ig increased with time (*P* = 0.017) but did not increase significantly by disease (*P* = 0.101). IgM increased with time (*P* < 0.001) and with disease neurological signs factor (*P* = 0.025) but an interaction between time and disease indicated a lower slope for neurologically diseased pigs (*P* = 0.038). IgG1 tended to increase by time (*P* = 0.058) but were not affected by the disease (*P* = 0.814). IgG2 were not increased by time but were lower in neurologically affected pigs than in controls (*P* = 0.027).

Total Ig, IgM, IgG1, and IgG2 reactive to the three *S. suis* isolates were analyzed and compared between isolates (see Supplementary Material). Total Ig and IgG2 levels differed among the three isolates being highest for isolate 109 (SS19), followed by 114 (SS2), and both being higher than isolate 115 (SS2) (*P* < 0.001; Supplementary Figure S1A and S1B). For IgG1, isolate 109 (SS19) had higher levels than 114 (SS2) and 115 (SS2). Otherwise, the levels of IgM were not significantly different between isolates (Supplementary Figure S1C).

## DISCUSSION

The present study brings insight about physiological changes occurring in meningitis affected naturally-infected pigs during *S. suis* outbreaks as well as the *S. suis* carrier status in tonsils and *S. suis* specific antibody levels. The monitoring of *S. suis* disease including the metabolic changes studied in the present study would be better controlled in a deliberate infection study; however, the present insights contribute to the understanding of the disease in practical conditions.

### The Disease and Tonsil Colonization

The diagnoses of outbreaks were based on clinical signs, necropsies in sudden death pigs and analysis of macroscopic lesions, histopathology, and *S. suis* culture (or DNA detection) in tissues. A SS19 was isolated once, and a SS2 was isolated twice, while SS2 and/or SS1/2, which cannot be differentiated on *csp2J* gene by Qpcr (Dekker, 2014), were detected by qPCR from tissues in all outbreaks. The SS19 which is not seen as a virulent serotype (Gottschalk and Segura, 2019) and was regarded a secondary infection to PRRSV which is endemic in the farm and is known to facilitate coinfections (Segura et al., 2020). The SS2 and/or SS1/2 represented the most frequently detected serotype in diseased pigs representing almost twice the prevalence in diseased pigs than control (81% vs. 44%). High loads of SS9 were also found in tonsils of neurologically affected pigs, but the relevance of this finding seems limited as none of the outbreaks was caused by SS9. Taking into account the anamnesis, the diagnosis of outbreaks, and the negative PCR to *Glaesserella parasuis* from tissues on the necropsied pigs, all evidence points out to *S. suis* SS2 as etiologicalagent, and we regard the current data as pathophysiology from such infection and meningitis.

Looking at the tonsil colonization, the prevalence of SS2 (and/or SS1/2) and its relatively abundance to total *S. suis* increased in neurologically diseased pigs, which was in alignment with the herd diagnosis. Furthermore, neurologically affected pigs showed higher number of sibling associations within the diseased group of pigs, which indicates that litter and sow origin were associated to both, a carrier status and a risk of suffering the disease. The transmission of virulent strains from the sow to piglets at birth (Amass et al., 1996) play a role on the disease and similarly would do the transmission of specific microbiota and its diversity (Niazy et al., 2022). Interestingly, diseased pigs had lower relative abundance of total *S. suis* than control ones. In one study, healthy pigs were found to have more *Bacteroides* and *Lachnospiraceae* in tonsils compared to tonsils from pigs confirmed with *S. suis* diseased (Niazy et al., 2022). It could hypothesized that control pigs are colonized with higher relative abundance of total *S. suis* species, and less susceptibility to be colonized by SS2. In another study, treated pigs with medium chain fatty acids + an anti-inflammatory had the highest diversity of nasal microbial and in turn showed the lowest *S. suis* disease prevalence (Correa-Fiz et al., 2020).

### Metabolic Changes

The disease reduced growth performance and implied some physiological changes as expected. Low-glucose levels in blood and reduced lipemia index, but increased icteric index in sera from diseased pigs were anticipated findings as expected with sepsis (Minemura et al., 2014). The jaundice observed may result either directly from bacterial products or as consequence from the host’s response to infection (Szabo et al., 2002). Similarly, meningitis increases anaerobic lactate production in BBB adjacent cerebral tissue by CSF pleocytosis and cellular infiltrates in the leptomeninges (Abassi et al., 2021). It is debated whether lactate production is associated to hypoxia (Møller et al., 2002). However, Buhr et al. (2021) recently validated that hypoxia is not occurring in CSF during *S. suis* induced meningitis using a catheter inserted into the subarachnoid space in pigs under anesthesia. The most widely accepted theory for increased lactate is that humoral factors are mediating the glycolysis metabolism in leptomeninges (de Almeida et al., 2011; Abassi et al., 2021). In bacterial meningitis, lactate in CSF is reported to increase about four to eight times (Sears et al., 1974; Guerra-Romero et al., 1992; Filho et al., 2014), which drops the pH and extensively compromises brain function as observed in the present study.

A pH drop in CSF has dramatic consequences, and the three-barrier BBB may become a disadvantage for ion exchange during meningitis as CSF is poorly buffered. The CO_2_ crosses the BBB better than HCO3^-^ and lactate (Weyne and Van Leuven, 1973); hence, the physiological response is to compensate with increased respiratory rate and reduce pCO_2_ from the blood. We measured ventilating rate at 120 respirations/min in one neurologically affected pig, while reference resting ventilation is expected at 25–40 respirations/min (Barbosa et al., 2019). Although we only measured respiratory rate once, a high ventilation rate is likely causing the reduced levels of CO_2_ and increased saturation of O_2_ and pH in blood observed. In other words, a partial respiratory alkalosis associated to meningitis acute acidosis in CSF. Further supporting this, the most acute case recorded (i.e., pig 96508) died 5 min after clinical detection and had an acute and severe acidosis (blood pH = 7.07 and BE = −11 mmol/L), which is opposite to the compensatory and respiratory alkalosis (average pH = 7.41) and low CO_2_ pressure in blood observed in the other diseased pigs and lower than the healthy pen-mates (pH = 7.3). The pCO_2_ in the CSF of rabbits with meningitis was reduced from 51 mm Hg (controls) to 37 mm Hg by hyperventilation and CSF pH fell below 7.20 only in one rabbit (Sears et al., 1974). In the present study, pigs with severe neurological signs lowered 11.3 mmHg pCO_2_ relative to control pen-mates (41.0 vs. 52.3 mm Hg pCO_2_). It can be speculated that surviving pigs may reach a stage where compensatory respiratory alkalosis occurs, while acute and poor prognosis pigs may suffer an acute metabolic acidosis often irreversible and found dead.

Another finding on the diseased pigs was the drop of minerals in blood. Meningitis can cause cerebral salt wasting syndrome and disrupt normal sympathetic system, ion exchange regulation, and antidiuresis with renal salt-wasting along the nephron (Bettinelli et al., 2012). Moderate and severe hyponatremia (serum Na < 130 mmol/L) were associated with disease severity, high incidence of cerebral edema, and increased risk of mortality in children with meningitis (Conner and Minielly, 1980; Chao et al., 2008). In the present study, the hyponatremia was moderate, and 50% of the neurologically affected pigs had serum Na between 130 and 136 mmol/L. Similarly, hypocalcemia (total and ionized) is common in children with severe meningococcal disease (Baines et al., 2000). Furthermore, low Mg concentrations may prevent parathormone secretion in response to hypocalcemia (Bushinsky and Monk, 1998) and contribute to the profile observed in the present study. The brain needs 48 h to adapt to a hypotonic environment, achieved mainly by extruding Na, Cl, K, other minerals, and organic osmoles from cells, while therapeutic corrections should not be too rapid (Sterns and Silver, 2006). Therapeutic concentrations of MgCl_2_ (500 mg/kg in animals and 2 mM in cultures) prevented pneumolysin‐induced brain swelling and tissue remodeling in brain slices (Hupp et al., 2017). Pneumolysin is a toxin from *S. pneumoniae* with a close relationship to *S. suis* suilysin (Segers et al., 1998). Perhaps, the Mg mechanism could perform similarly in streptococcal meningitis. We encourage to research electrolyte technologies, mineral supplementation, and hydration as recovery program for *S. suis* diseased pigs which could help reduce mortality and recovery time.

### Antibody Status

The age at sampling was positively associated with increasing *S. suis* reactive total Ig and IgM levels in serum as expected. On the other hand, total Ig, IgG1, and IgG2 levels differed depending on the isolate used as antigens, with a higher reactivity of IgG2 toward SS19 isolate compared to the two SS2 isolates. The difference could be explained by the level of CPS expression (Dolbec et al., 2023) and/or the immunological properties of surface proteins specific to each strain (Okura et al., 2021), including the presence or not of cross-reacting antigens. (Okura et al., 2021). As aforementioned, the relative contribution of IgG1 and IgG2 to *S. suis* clearance is still unclear (Butler and Brown, 1994). Nevertheless, a recent study indicated that swine IgG1, IgG2a, IgG2b, IgG2c, and IgG4 bind well to targeted cell types and mediate antibody-mediated cell phagocytosis (Paudyal et al., 2022), suggesting a potential role for these isotypes to eliminate *S. suis*—a hypothesis that remains to be verified and requires a different study design. Interestingly, neurologically diseased pigs showed higher levels of *S. suis* reactive IgM with a lower slope over time. Rieckmann et al. (2018), observed that pigs in commercial settings go through an early adaptive immune IgM response against *S. suis* between 4.5 weeks and 6–8 weeks postweaning even without detectable clinical signs, which aligns with the current findings. However, little data are available for IgM kinetics in naturally diseased by *S. suis.* From *Streptococcus pneumonia*, it is known that IgM can be produced very early, i.e., 3 d, during the extrafollicular phase of the adaptive immune response (Koppel et al., 2008). Therefore, IgM results could indicate that diseased pigs were exposed earlier to *S. suis* than control and before presenting clinical signs. Furthermore, the higher levels of reactive IgM observed in diseased pigs could be the result of cross-reactions and memory recall with other commensal bacterial species, including other streptococci.

## CONCLUSION

In conclusion, occurrence of *S. suis* disease in nursery pigs was associated to average performing pigs without any predisease trait to highlight but a sow/litter effect. The neurologically affected pigs showed increased SS2 (and/or 1/2) prevalence and relative abundance in tonsils, lower *S. suis* reactive IgG2 and higher IgM in serum. Besides, diseased pigs showed metabolic changes including a respiratory alkalosis and loss of minerals. Further investigations to support the pH changes, water loss and mineral recovery in diseased pigs are warrantied.

## Supplementary Material

txad126_suppl_Supplementary_Tables_S1-S2_Figures_S1
