# Supplementary material for: Metabolic insights and background from naturally affected pigs during Streptococcus suis outbreaks
Source: Transl Anim Sci. 2023 Nov 6;7(1):txad126. doi: 10.1093/tas/txad126 (PMC10660374; doi:10.1093/tas/txad126)
Supplement: txad126_suppl_Supplementary_Tables_S1-S2_Figures_S1 [file txad126_suppl_supplementary_tables_s1-s2_figures_s1.docx]

**Table S1**. Retrospective information for sow and litter origin from diseased pigs, neurologically diseased pigs, and controls.

|  | **All clinical signs** | | | |  | **Neurological signs** | | | |
| --- | --- | --- | --- | --- | --- | --- | --- | --- | --- |
|  | **Control** | **Diseased** | RMSE | P-value |  | **Control** | **Diseased** | RMSE | P-value |
| Sow parity | 4.03 | 5.00 | 2.74 | 0.136 |  | 4.03 | 5.15 | 2.87 | 0.187 |
| Total born piglets, n | 15.8 | 15.2 | 4.12 | 0.561 |  | 15.8 | 14.4 | 4.18 | 0.269 |
| Born alive piglets, n | 14.8 | 14.3 | 4.03 | 0.598 |  | 14.8 | 13.7 | 4.03 | 0.355 |
| Stillborn piglets, n | 0.97 | 0.89 | 1.12 | 0.812 |  | 0.97 | 0.70 | 1.04 | 0.387 |
| Mummified  piglets, n | 0.21 | 0.43 | 0.81 | 0.328 |  | 0.21 | 0.40 | 0.77 | 0.390 |
| Av. birth weight, kg | 1.44 | 1.53 | 0.31 | 0.256 |  | 1.44 | 1.58 | 0.32 | 0.116 |
| Litter mortality, n | 1.76 | 1.36 | 1.70 | 0.312 |  | 1.76 | 1.10 | 1.55 | 0.151 |
| Litter mortality, % | 11.58 | 8.49 | 10.6 | 0.220 |  | 11.58 | 7.18 | 9.82 | 0.130 |
| Weaned, n | 13.3 | 13.6 | 0.95 | 0.266 |  | 13.3 | 13.6 | 0.98 | 0.405 |
| Litter average BW at  weaning, kg | 6.79 | 6.64 | 1.13 | 0.613 |  | 6.79 | 6.69 | 1.19 | 0.766 |

^1^Root mean standard error.

**Table S2.** Blood parameters and serum minerals between all diseased and controls pigs and between neurologically diseased only and control pigs.

|  | **All clinical signs** | | | |  | **Neurological signs** | | | |
| --- | --- | --- | --- | --- | --- | --- | --- | --- | --- |
|  | **Control** | **Sick** | RMSE | P-value |  | **Control** | **Sick** | RMSE | P-value |
| Number of pigs | *28* | *28* |  |  |  | *28* | *20* |  |  |
| pH (blood) | 7.30 | 7.41 | 0.09 | <.0001 |  | 7.30 | 7.41 | 0.10 | <.001 |
| PCO_2_ (blood), mmHg | 52.5 | 41.9 | 9.34 | <.001 |  | 52.5 | 41.0 | 9.86 | <.001 |
| PO_2_ (blood), mmHg | 35.5 | 38.3 | 11.01 | 0.365 |  | 35.5 | 39.4 | 11.7 | 0.258 |
| Base Excess (blood), mmol/L | -1.39 | 1.50 | 4.58 | 0.022 |  | -1.39 | 1.40 | 4.88 | 0.062 |
| HCO_3_^1^ (blood), mmol/L | 25.2 | 26.2 | 3.86 | 0.380 |  | 25.2 | 26.0 | 4.10 | 0.574 |
| TCO_2_^1^ (blood), mmol/L | 26.9 | 27.5 | 3.99 | 0.571 |  | 26.9 | 27.2 | 4.24 | 0.807 |
| sO2^1^ (blood), mmol/L | 57.1 | 67.4 | 16.2 | 0.023 |  | 57.1 | 67.6 | 17.2 | 0.045 |
| Na (blood), mmol/L | 139 | 137 | 3.37 | 0.044 |  | 139 | 137 | 3.27 | 0.008 |
| K (blood), mmol/L | 5.21 | 4.53 | 0.78 | 0.002 |  | 5.21 | 4.49 | 0.79 | 0.004 |
| iCa (blood), mmol/L | 1.38 | 1.30 | 0.07 | <.0001 |  | 1.38 | 1.27 | 0.07 | <.0001 |
| Glucose (blood), mg/L | 117.5 | 88.7 | 21.5 | <.0001 |  | 117.5 | 82.3 | 21.5 | <.0001 |
| Hematocrit, % PCV | 31.8 | 30.6 | 3.82 | 0.226 |  | 31.8 | 30.4 | 3.19 | 0.155 |
| Hemoglobin^1^, g/dL | 10.8 | 10.4 | 1.31 | 0.242 |  | 10.8 | 10.3 | 1.10 | 0.163 |
| Acidosis BE < -3 (0) vs. Alkalosis BE > +3 (1) | 0.219 | 0.756 | 0.13^2^ | 0.026 |  | 0.219 | 0.731 | 0.15^2^ | 0.038 |
| Acidosis BE < -3  (yes = 1, no = 0) | 0.359 | 0.107 | 0.09^2^ | 0.040 |  | 0.359 | 0.153 | 0.10^2^ | 0.156 |
| Alkalosis BE > +3  (yes = 1, no = 0) | 0.107 | 0.325 | 0.10^2^ | 0.064 |  | 0.107 | 0.422 | 0.13^2^ | 0.019 |
| Ca (serum), mmol/L | 2.59 | 2.35 | 0.15 | <.0001 |  | 2.59 | 2.33 | 0.15 | <.0001 |
| K (serum), mmol/L | 4.97 | 4.55 | 0.72 | 0.038 |  | 4.97 | 4.5 | 0.71 | 0.026 |
| Mg (serum), mmol/L | 0.911 | 0.866 | 0.08 | 0.035 |  | 0.911 | 0.871 | 0.07 | 0.057 |
| P (serum), mmol/L | 2.91 | 2.69 | 0.39 | 0.039 |  | 2.91 | 2.69 | 0.36 | 0.044 |
| Icterie index (serum) | 0.286 | 1.89 | 1.55 | <.001 |  | 0.286 | 2.25 | 1.40 | <.0001 |
| Hemolysis index (serum) | 7.68 | 2.32 | 7.06 | 0.006 |  | 7.68 | 2.7 | 7.51 | 0.029 |
| Lipemia index (serum) | 20.8 | 11.5 | 7.03 | <.0001 |  | 20.8 | 9.9 | 6.65 | <.0001 |

^1^Calculated values on iSTAT® equipment (Abbott Point of Care Inc., Princeton, NJ, USA)

^2^Standard error of the mean.

**Figure S1.** Serum levels (log2 ± SEM) of total Ig (A), IgM (B), IgG1 (C), and IgG2 (D) reactive to three *Streptococuss suis* specific isolates determined serotypes (SS) 2 and 19 including age at sampling as covariate.

^a–b^Different superscripts indicate a significance between categories (P < 0.05).
